# JNK3 inhibitors as promising pharmaceuticals with neuroprotective properties

**DOI:** 10.1080/19336918.2024.2316576

**Published:** 2024-02-15

**Authors:** Yibeini Wu, Yiling Zhao, Ziman Guan, Sajjad Esmaeili, Zhicheng Xiao, Diji Kuriakose

**Affiliations:** a Department of Anatomy and Developmental biology, Monash University, Clayton, Vic, Australia; b Shaoxing Institute, Zhejiang University, Shaoxing, China

**Keywords:** Apoptosis, inhibitor, JNK3, MAPK signalling pathway, neurodegenerative disease, neuroinflammation, neuroprotection, oxidative stress

## Abstract

The intensive study and investigation of neuroprotective therapy for central nervous system (CNS) diseases is ongoing. Due to shared mechanisms of neurodegeneration, a neuroprotective approach might offer benefits across multiple neurological disorders, despite variations in symptoms or injuries. C-Jun N-terminal Kinase 3 (JNK3) is found primarily in the CNS and is involved in physiological processes such as brain development, synapse formation, and memory formation. The potential of JNK3 as a target for pharmacological development holds promise for advancing neuroprotective therapies. Developing small molecule JNK3 inhibitors into drugs with neuroprotective qualities could facilitate neuronal restoration and self-repair. This review focuses on elucidating key neuroprotective mechanisms, exploring the interplay between neurodegenerative diseases and neuroprotection, and discussing advancements in JNK3 inhibitor drug development.

## Introduction

Neuroprotective therapy is a treatment for diseases of the CNS that is being researched and explored comprehensively. Neuroprotection can help people suffering from diseases or brain injuries that affect the nervous system, including stroke, multiple sclerosis and neurodegenerative diseases to name a few [1]. Some approaches to neuroprotection may be helpful for more than one neurological disorder because many of the mechanisms behind neurodegeneration are the same, despite differences in symptoms or injuries. Current neuroprotective agents can reverse some of the existing damage, and they can slow the degeneration of the CNS and the progression of the disease by stopping or preventing further neurological damage [2]. Researchers are also looking for ways to block or delay apoptosis by protecting nerves after acute events such as cerebral ischemia.

The use of JNK3 as a potential target is one possible approach to neuroprotective therapy. JNK signaling is an important factor in induced apoptosis. Three different genes encode the three JNK isoforms, JNK1, JNK2, and JNK3. The expressions of JNK1 and JNK2 are found all over the body. On the other hand, JNK3 is observed specifically and strictly in the CNS [3]. Therefore, if we could find drugs that strictly bind to JNK3, it would have fewer side effects. JNK3 is a multifunctional enzyme that can be seen as a signaling sensor for neurodegeneration in pathological conditions. JNK3 controls both neuronal and non-neuronal cell physiological functions including development, differentiation, regeneration, and reprogramming. Given the specific location and function of JNK3 in the CNS, the use of JNK3 as a potential target for pharmacological development may be a possibility for neuroprotective therapies [4]. This review aims to discuss the main neuroprotective mechanisms, associated with neurodegenerative diseases, and also the development of small molecule JNK3 inhibitors.

## Neuroprotection and neurodegenerative diseases

**Figure 1.**
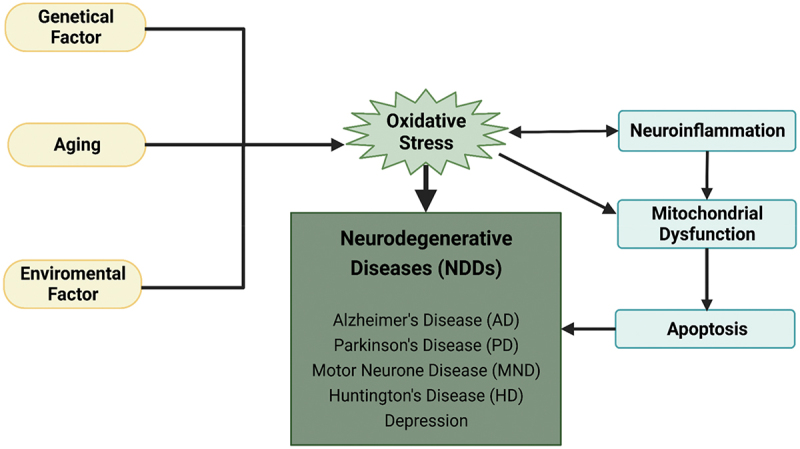
The overall schematic representation of neurodegenerative diseases, including common factors, NDDs and mechanisms [created by BioRender].

Neurodegenerative diseases, such as Alzheimer’s disease (AD), Parkinson’s disease (PD) and Motor Neurone Disease (MND), are age-related progressive diseases characterized by the degeneration of selectively susceptible populations of neurons in specific areas in the CNS. As shown in Figure 1, genetical factor, aging, environmental factor are the main risk factors in neurodegenerative disease. Postmitotic cells such as neurons are lifelong [5]. As a result, organisms and cells have evolved self-repair mechanisms which enable them to live for a long period of time. It shows that neuroprotection may have effects that lead to the function and structure of the CNS to restore, recover and regenerate [6].

In recent years, the discovery process for neuroprotectors has centered on preventing the pathophysiological processes that cause a neuronal loss in neurodegeneration. However, only a small number of the strategies evaluated in some studies were effective in slowing or preventing neurodegeneration. Endorsing neuroprotection mechanisms, also known as cellular resilience, help in neuronal regeneration and self-healing. Despite these mechanisms have not obtained adequate attention, these ways still open up new therapeutic possibilities for preventing neuronal death and mitigating neurodegeneration [7]. We describe the primary neuroprotective mechanisms and how they contribute to the survival of neurons during neurodegeneration.

Protein kinases known as mitogen-activated Protein Kinases (MAPKs) control both basic biological functions and cellular reactions to stress signals from the outside external environment. In mammalian cells, the MAPK pathway can transmit, amplify, and integrate signals from various stimuli, eliciting appropriate physiological responses such as development, apoptosis, proliferation, inflammatory response and differentiation [8]. Potential targets for anti-inflammatory therapy, MAPKs – particularly p38 MAPK – have enhanced activity and are regulated by the production of inflammatory cytokines at the transcriptional and translational levels [9]. While the Akt protein kinase B (PI3K-Akt) and phosphatidylinositol 3-kinase and MAPK/Erk1,2-pathways are significant signaling cascades that regulate neuronal development, survival, and differentiation the JNK and p38 MAPK pathways have been associated with cellular stress and apoptosis, as well as neuronal protection [10]. The intricate coordination of molecular pathways governing c-Jun N-terminal Kinase 3 (JNK3) activation in neuronal diseases constitutes a sophisticated interplay of signaling cascades responsive to diverse cellular stressors. The activation of JNK3 is notably linked to oxidative stress, neuroinflammation, and the accumulation of misfolded proteins, all implicated in the pathogenesis of neurodegenerative diseases. Crucially, upstream kinases, notably mitogen-activated protein kinase kinases (MKK4 and MKK7), assume a pivotal role in phosphorylating and activating JNK3 [11]. Moreover, the participation of mitogen-activated protein kinase kinases (MAP3Ks) initiates a cascade culminating in JNK3 activation, thereby contributing to an intricate regulatory network. This activation paradigm, intricately intertwined with other signaling pathways, emphasizes the multifaceted nature of JNK3’s involvement in neuronal diseases. A comprehensive understanding of these molecular intricacies is essential for formulating targeted therapeutic strategies aimed at alleviating JNK3-associated neurodegenerative processes [12].

The mechanism by which JNK3 inhibitors operate revolves around pinpointing specific elements within the JNK signaling pathway to hinder its activation. Reversible inhibitors typically vie with ATP for attachment to JNK3’s catalytic site, thereby hindering substrate phosphorylation. In contrast, irreversible inhibitors establish covalent bonds with JNK3, ensuring prolonged inhibition. Furthermore, certain inhibitors focus on upstream kinases, disrupting the sequential activation cascade [13]. The detailed intricacies of these inhibitory processes offer readers valuable insights into potential therapeutic approaches, underscoring the requisite precision for efficacious JNK3 modulation in neuroprotective interventions.

### Mechanism of neuroprotection in neuroinflammation

Neuroinflammation, driven by specific or nonspecific immune responses initiated by microglial stimulation in the CNS, plays a pivotal role in neuronal protection. Microglia, serving as sentinels for neurons with limited regenerative capacity, maintain immunological homeostasis by clearing debris, eliminating pathogens, and orchestrating immune responses. However, in the aftermath of brain injury or the onset of neurodegenerative diseases, activated microglia release neurotoxins and cytokines, including tumor necrosis factor (TNF-α), interleukin 6 (IL-6), IL-1, and nitric oxide, contributing to inflammation and potentially accelerating neuronal degeneration [14].

A critical facet of this neuroinflammatory cascade involves the bidirectional interplay between JNK3 phosphorylation and the activation of inflammatory cytokines. The phosphorylation of JNK3 is intricately linked to the activation of inflammatory cytokines, forming a feedback loop that perpetuates neuroinflammation. Conversely, the activation of inflammatory cytokines can phosphorylate JNK3 within neurons, establishing a reciprocal relationship. In preclinical models of neurodegenerative diseases, innovative drugs with anti-inflammatory effects intervene in this intricate process by blocking the expression of inflammatory mediators and modulating the p38 MAPK and JNK signaling cascades [9].

The targeted modulation of these pathways not only reduces pro-inflammatory cytokines but also exhibits promise in shielding neurons from neurotoxicity. Additionally, the PI3K-Akt and MAPKs pathways, crucial for neuronal survival, offer diverse protective properties in neurons and glial cells associated with central nervous system diseases [15]. The activation of inflammatory cytokines, characterized by their role in initiating and perpetuating neuroinflammation, further emphasizes the complex interplay between immune responses and JNK3 phosphorylation, providing a nuanced understanding of neuroprotection mechanisms in the context of neuroinflammation.

### Mechanism of activation of microglia

An insightful investigation into the mechanisms underlying microglial activation by JNK3 is exemplified in a study on indirect traumatic optic neuropathy (ITON). ITON triggered the activation of the NLRP3 inflammasome and a subsequent microglial response, primarily mediated by JNK/c-Jun signaling. The study focused on the neuroinflammatory cascade’s contribution to the degeneration of retinal ganglion cells (RGCs), a critical factor in ITON-associated permanent vision loss following blunt head trauma [16]. Detailed results from the investigation revealed significant RGC death and axonal degeneration in the ITON model, concurrently activating the JNK/c-Jun signaling pathway. This activation, in turn, elicited a robust microglial response and NLRP3 inflammasome activation. Notably, disruption of the JNK pathway demonstrated its pivotal role in suppressing NLRP3 inflammasome activation in microglia, thereby preventing RGC death and axonal degeneration [17,18].

The findings further emphasized the role of JNK/c-Jun signaling in ITON, illustrating its capacity to activate the NLRP3 inflammasome in microglia, thereby contributing to axonal degeneration and RGC demise. Crucially, the study suggests JNK inhibition as a promising strategy to effectively suppress the inflammatory response, offering a potential avenue to enhance RGC survival [19]. While additional research is warranted to explore the translational potential of pharmacological NLRP3 inflammasome inhibition in preventing ITON, these findings provide promising insights for future therapeutic interventions.

### Mechanism of neuroprotection in apoptosis

Several experimental models have highlighted the neuroprotective benefits of certain drugs, demonstrating improvements in neurological dysfunction. These drugs effectively reduce excess reactive oxygen species (ROS), enhance in vitro oxygen-glucose deprivation (OGD), and activate the PI3K/AKT and MAPK pathways to inhibit apoptosis. In this context, they elevate AKT and extracellular signal-regulated kinase (ERK) levels while decreasing the phosphorylation of JNK and p38, ultimately shielding neurons from apoptosis [20,21].

Apoptosis, a vital process in programmed cell death (PCD), intricately contributes to various pathological conditions, including neurological disorders. Recent studies shed light on the multifaceted roles of caspases, traditionally associated with cell death, in remodeling the nervous system without causing cell demise in neurodegenerative tissues [22].

Critical anti-apoptotic pathways, regulated by FLICE-inhibitory proteins, Bcl-2, and Inhibitors of Apoptosis Proteins (IAPs), serve as essential safeguards against apoptosis. IAPs, particularly XIAP, are implicated in neuroprotection, and alterations in their function are linked to Parkinson’s disease etiology. IAPs also contribute to ischemic preconditioning, enhancing cell survival after caspase cascade activation [23]. Additionally, pro-survival actions of glial cell-derived neurotrophic factor (GDNF) and molecular pathways like extracellular signal-regulated kinases (ERK) and AKT play pivotal roles in preventing cell death.

The AKT pathway, recognized as a pro-survival factor, inhibits apoptosis inducers like p53, finely modulating the balance between cell survival and death. FOXO transcription factors, phosphorylated by AKT, exhibit dual roles – promoting cell survival when altered and connecting to apoptosis when activated. Manipulating FOXO family members provides a novel avenue to enhance neuronal survival by inhibiting apoptosis [24].

Furthermore, heightened neuronal activity, attributed to increased expression of NMDA-dependent anti-apoptotic genes, emerges as a robust promoter of anti-apoptosis. These genes enhance mitochondrial stress resistance, fortifying cells against injury. This intricate network of apoptotic regulation and anti-apoptotic mechanisms offers a nuanced understanding of neuronal survival strategies, presenting potential targets for therapeutic interventions [25].

### Mechanism of neuroprotection in oxidative stress

An imbalance in the generation and accumulation of ROS in cells and tissues, as well as in the capacity of biological systems to remove these ROS byproducts, results in oxidative stress. As byproducts of oxygen metabolism, ROS play an important role in a variety of physiological conditions under normal circumstances. The generation of ROS is necessary for a variety of biological processes, such as the activation of numerous transcription factors, protein phosphorylation, apoptosis, cell differentiation and immunity. However, the level of intracellular ROS should be kept low. Because of its high oxygen demand and the existence of peroxide-sensitive cells, the brain is the organ most sensitive to ROS [26]. Several research has revealed that oxidative stress is critical in the progression of neurodegenerative diseases.

A potential therapeutic strategy that might lessen the apoptosis brought on by oxidative stress is the development of medications with specific neuroprotective effects. Several medications have been found to improve endogenous antioxidant defense mechanisms, which are participated in neuroprotection via the Nrf2-ERK1/2 MAPK pathway, and scavenge high levels of ROS to combat excessive ROS [15].

## JNK3 and neuroprotection

### The biological characteristic of the JNK3

**Figure 2.**
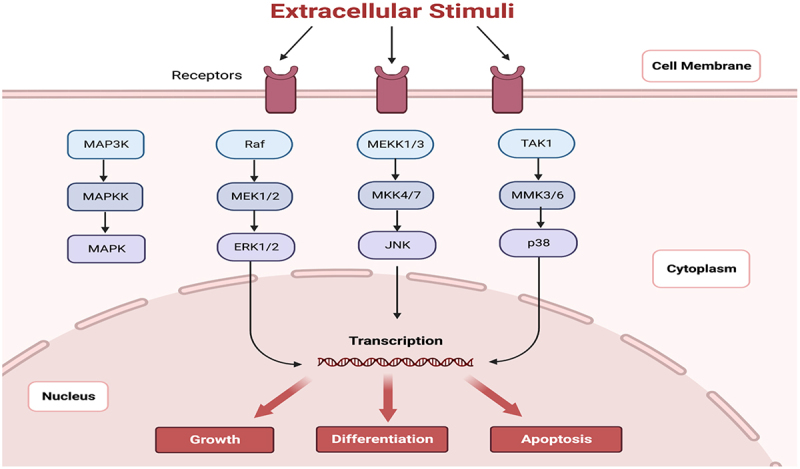
MAPK and JNK kinase signalling pathways with extracellular stimuli to receptors and trigger multiple signalling pathways MAP3K, Raf, MEKK1/3 and TAK1, lead to transcription in nucleus [created by BioRender].

**Figure 3.**
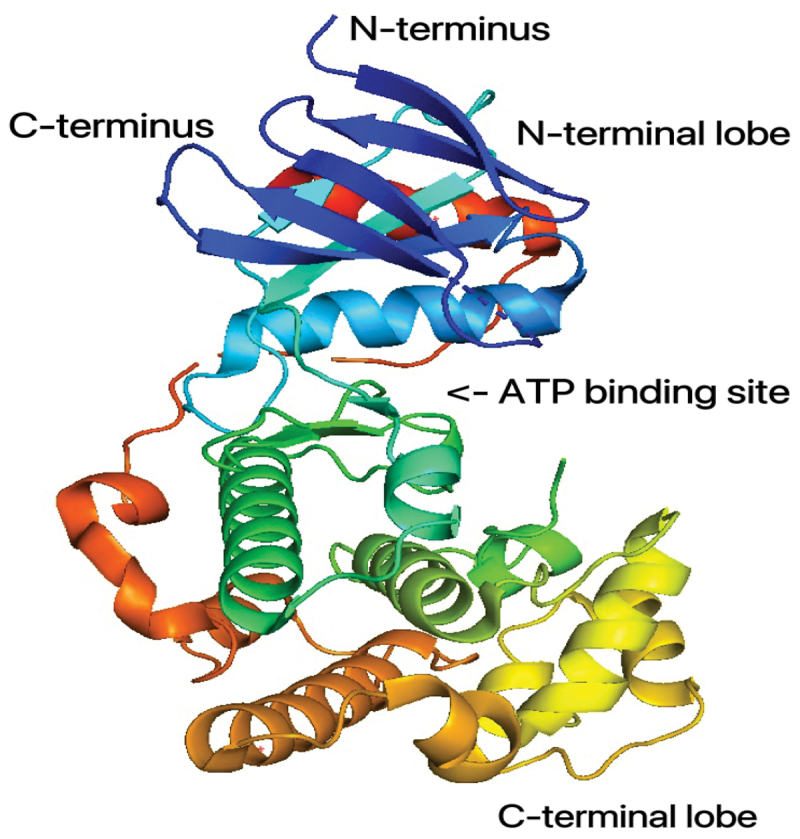
The 3D crystal structure of JNK3 with ATP binding site. (PDB no. 2b1p) [30]. The structure is illustrated in ribbon representation, with colours changing from blue through green and yellow to red from the N terminus to the C terminus.

C-Jun NH 2-terminal kinase, also known as JNK, is a protein kinase that, along with ERK and MAPK p38, belongs to the family of enzymes known as mitogen-activated protein kinases (MAPKs) [27]. As illustrated in Figure 2, MAPKs are known to play a role in the regulation of a variety of cellular functions and Activation of c-Jun can be induced by JNK, and the phosphorylation and continued expression of c-Jun is a necessary step in the pathway that leads to the induction of apoptosis [28]. To date, three identically structured isoforms of JNK have been identified in humans, namely JNK1, JNK2, and JNK3. JNK1 and JNK2 are two of these proteins that have the potential to be expressed in a wide variety of tissues throughout the body [29]. It is noteworthy to mention that only JNK3 is expressed at high levels in the central nervous system, despite the fact that JNK3 shares 92% and 87% of its amino acid homology with JNK1 and JNK2, respectively (brain) [30]. Using its crystal structure as shown in Figure 3, Xie et al. demonstrated in 1998 that JNK3 has a typical kinase fold structure. This means that it has an ATP binding site sandwiched between two sub-structural domains of the catalytic domain (the N-terminal domain and the C-terminal domain), as well as a non-catalytic domain that enables substrate attachment and the recruitment of other signaling proteins. In addition, a comparison of the amino acid sequences showed an additional substrate binding site at the c-terminus of JNK3, separate from JNK1 and JNK2, which is important for studying the specific binding of JNK3 to specific substrates [31].

### JNK3 is a potential therapeutic target for neurological disorders

In humans, JNK1 and JNK2 can be found in all kinds of tissues, but JNK3 is mostly only found in the brain, heart, testis, and islets [29]. As illustrated in Figure 1, JNK3 has been demonstrated to be most sensitive to many stressful stimuli in the CNS, such as ischemia, oxidative stress, apoptosis, inflammation, and Aβ oligomer toxicity [32]. Moreover, because JNK3 levels may be measured at peripheral levels, JNK3 can be employed as a biomarker for certain neurodegenerative and neurodevelopmental illnesses, as well as a therapeutic development target [11]. Kuan et al. discovered in 2003 that the targeted absence of JNK3 could not only activate JNK activity by stress but also protect mice from brain damage caused by cerebral ischemia and hypoxia, indicating that JNK3 was a promising target for the development of neuroprotective therapeutics for stroke [3]. In addition, Mathis and colleagues discovered that the expression level of JNK3 in the central nervous system was substantially connected with the pathological scores of amyloid plaques (AP) and neurofibrillary tangles (NFT) [33]. They also discovered that JNK3 was directly implicated in the phosphorylation of APP and Tau, two Alzheimer’s disease (AD) indicators [33]. This indicates that the specific expression of JNK3 in the brain is essential for the CNS and has the potential to be a target for AD diagnosis and targeted drug development. In a subsequent study conducted in 2015, Jing et al. discovered that apoptotic signal-regulated kinase 1 (ASK1) is a positive regulator of JNK3 and that corresponding stress stimulation can activate the ASK1-JNK3 signaling pathway in brain cells [34]. ASK1-JNK3 signaling pathway plays a key role in the pathogenesis of Parkinson’s disease (PD), and activated JNK3 can induce apoptosis by phosphorylating a variety of target proteins [34], so both ASK1 and JNK3 are potential targets for the development of PD treatment. Liu et al. also found that after optic nerve axotomy (ONA), JNK3 would be upregulated and contribute to Tuj1^+^ RGC (retinal ganglion cell) death. This pathway may be another novel target for neuronal protection [35].

In addition, Yang et al. found that JNK3 knockout mice showed decreased apoptosis of hippocampal nerve cells, and the frequency of epileptic symptoms in such mice was reduced after glutamate receptor agonist induction [36]. Li et al. indicated that JNK3 knockout mice have reduced levels of Nav1.6 sodium channel cell surface expression in the white matter of the spinal cord and spinal conduction velocity, which means JNK3 could regulate neuronal excitability by regulating the surface expression of sodium channels [37]. Therefore, JNK3 can be regarded as a potential target for the treatment of some neurological diseases. And the reduction of JNK3 activity, the targeted deletion of JNK3, and the knockout of the JNK3 gene can all have certain effects on the alleviation of related neurological illnesses.

## The development of JNK3 inhibitors

In recent years, researchers have found that the creation of inhibitors that target JNK3 is a very attractive strategy for treating neurodegenerative diseases as well as a direction to investigate neuroprotective approaches. To date, some compounds that inhibit JNK3 activity and biological function have shown promise as neuroprotective drugs, and these JNK3 inhibitors shown in Table 1 can be divided into two groups, irreversible and reversible, based on how tightly they bind to the protein.

**Table 1.** Different reversibility inhibitors and irreversibility inhibitors exert neuroprotective effects in different ways and related effect of action.

Compound	Type	Effect of Action	References
JNK-IN-8	Irreversible Inhibitor	reduce the activation of microglia and inhibit neuroinflammation	[38,39]
FMU200	Irreversible Inhibitor	Reduce RPS production and TNF-α level	[40]
SP600125	Reversible Inhibitor	Inhibit neuron apoptosis	[41,42]
SP6001245	Reversible Inhibitor	Reducing synaptic damage and improve long-term memory capacity	[43]
AS602801 (Bentamapimod)	Reversible Inhibitor	Inhibit JNK phosphorylation and caspase-3 expression	[44]
Amino Pyrazole Derivatives	Reversible Inhibitor	Inhibit cell death and ROS production	[45]
IQ-1S	Reversible Inhibitor	Reverse cerebral ischemia infarction	[46]
PT 109	Reversible Inhibitor	Reduce ROS and inflammatory factors	[47]
thiophene-pyrazoleuronium derivatives	Reversible Inhibitor	Balance Brain Penetration	[48]
N-aromatic substituted indazole derivatives	Reversible Inhibitor	Balance Brain Penetration	[49]

### Irreversible JNK3 inhibitors

Typically, irreversible JNK3 inhibitors combine with groups in JNK3 with relatively strong covalent bonds to achieve the effect of losing its enzymatic activity. As early as 2012, Zhang et al. found that JNK-in-8, which is a benzamide derivative, is a potent, specific, and irreversible intracellular inhibitor of JNK activity. They reached this conclusion by conducting extensive biochemical and cell-selective analysis and found that JNK-IN-8 can interact with JNK3 by forming a covalent bond [38]. After seven years, Zheng et al. discovered that JNK-IN-8 could exert anti-inflammatory effects by reducing the activation of microglia and the expression of cellular inflammatory factors (IL-6, IL-1, and TNF-α). They also discovered that it could inhibit neuroinflammation and improve neurological function by inhibiting the JNK/NF-KB signaling pathway, and it could achieve a neuroprotective effect [38,39]. Additionally, in the year 2016, Muth and colleagues identified that the pyridyl imidazole derivative FMU200 acts as a covalent inhibitor of the JNK3 protein [40]. Later, in 2021, Stephanie and colleagues showed that FMU200 could reduce the production of ROS and the content of inflammatory factor TNF-α in neuroblastoma SH-SY5Y cells by inhibiting JNK activity [41,42], thus playing an antioxidant and neuroprotective effect.

### Reversible JNK3 inhibitors

Reversible JNK3 inhibitors are those that have the effect of lowering the activity level of JNK3 by binding to JNK3 using non-covalent bonds that are not as stable. Among the reversible JNK3 inhibitors that have been identified, the ones that bind to the ATP-binding site of JNK3 in a competitive manner are the ones that have garnered the most attention from scientists. In 2006, Guan et al. reported that SP600125 is a powerful JNK inhibitor that is also selective and cell-permeable and that its effects can be reversed [42]. It was the first JNK inhibitor that was obtained through synthetic means, and it was also a pan-inhibitor, which means that it exhibited potent inhibition of JNK1, JNK2, and JNK3 (IC50: 40/40/90 nM) [42]. In a series of histological analyses and biochemical tests, Guan et al. discovered that SP600125 had the ability to inhibit JNK3 by binding to the ATP-binding site in a manner that was competitive [42]. This prevented the activation of the JNK signaling pathway, which led to the achievement of apoptosis inhibition and neuroprotection [42]. Subsequently, in 2008, Carboni et al. demonstrated that another ATP-competitive JNK pan-inhibitor, AS6001245 (the IC50 of JNK1/JNK2/JNK3 is 150/220/70 nM), could improve long-term memory capacity in gerbils by inhibiting JNK activity. This was accomplished by reducing synaptic damage [43]. Later, in 2016, Chen et al. demonstrated through their study that a JNK pan-inhibitor Bentamapimod (AS602801, the IC50 of JNK1/JNK2/JNK3 are 80/90/230 nM) can also inhibit JNK phosphorylation and activity by competitively binding to the ATP-binding site of JNK3 expression of Caspase-3 protein (a common marker of apoptosis) to attenuate neuronal injury [44]. Cheng et al. proposed the new and selective JNK3 binder scaffold 5-(phenylamino)-1 H–1,2,3-triazole-4-carboxamide by using fluorescence thermal shift screening, native mass spectrometry and molecular docking. They provided an HTS-FTS tool for quickly identifying new JNK3 inhibitor scaffolds [50].

There are several other reversible JNK3 inhibitors that have neuroprotective effects, in addition to the inhibitors that were mentioned above that bind the ATP-binding site in JNK3 in a competitive manner. These include, among other things, a series of amino pyrazole derivatives identified by Zheng et al. in 2014 as being able to bind JNK3 through the formation of hydrogen bonds. By inhibiting JNK3 activity, these compounds exert a neuroprotective effect by preventing the death of neuroblastoma SH-SY5Y cells induced by 6-OHDA and reducing the production of reactive oxygen species [45]. Later, in the year 2020, Plotnikov et al. discovered a novel high-affinity JNK3 inhibitor called 11 H -indeno[1,2- b]quinoxalin-11-one oxime sodium salt (IQ-1S). The IQ-1S can bind to JNK3 by forming two hydrogen bonds, and this inhibitor shows an obvious dose-dependent neuroprotective effect in the rat model of transient focal cerebral ischemia [46]. In the same year, Chen et al. discovered that a novel small molecule, PT109, can bind to JNK3 and inhibit its activity by forming four hydrogen bonds. Additionally, they found that PT109 demonstrates anti-neuroinflammatory and neuroprotective effects by promoting hippocampal neurogenesis, reducing the accumulation of ROS, and decreasing the levels of inflammatory factors (TNF- α, IL-β, IL-6) [47]. One year later, Feng et al. found the thiophene-pyrazoleuronium derivatives and the N-aromatic substituted indazole derivatives that could bind to JNK3 by hydrogen bonds and suppress its activity. And both these two kinds of derivatives can exhibit some protective effects on the CNS by balancing brain penetration [48,49].

### Safety and efficacy of JNK3 inhibitors

The safety profile of JNK3 inhibitors has been a focus of rigorous investigation, employing meticulous in vivo testing to ascertain both safety and efficacy. Multiple preclinical studies utilizing animal models have aimed to elucidate safety parameters, encompassing potential adverse effects, toxicity levels, and overall safety profiles associated with the administration of JNK3 inhibitors. These studies employ sophisticated methodologies to ensure the reliability and relevance of the obtained data. Recent research on various inhibitors, including JNK3, has significantly advanced our understanding of the signaling cascade in diverse neurodegenerative models [51]. Bentamapimod, an orally active pan-JNK inhibitor, demonstrated a favorable safety and toxicity profile in phase I and II clinical trials for inflammatory endometriosis, encompassing safety and pharmacokinetics in phase I and clinical efficacy evaluation for ocular inflammation and pain associated with cataract surgery in phase III [52]. In safety assessments, CC-930, a potent and selective JNK inhibitor, exhibited no in vitro toxicity, did not inhibit CYP450 enzymes, and was well-tolerated in healthy male volunteers. However, its phase II clinical trial for idiopathic pulmonary fibrosis was terminated due to cardiac adverse effects [53]. AGI-1067, inhibiting TLR4 ligand-induced activation of ASK1 and downstream p38 and JNK MAP kinases, has been assessed for safety and efficacy in treating vascular inflammation and atherosclerosis in cardiovascular events [54]. These diverse studies contribute valuable insights into the safety profiles and potential clinical applications of JNK3 inhibitors.

### Exploration of natural compounds for JNK3 inhibition

The exploration of natural compounds holds immense promise in the pursuit of new and effective drugs, with the advantage of fewer side effects compared to many synthetic substances. Over the past several decades, the interest in naturally occurring compounds has surged as researchers seek potent drugs with enhanced safety profiles, especially those diseases mediated by protein kinases.

In addition to chemically synthesized molecules, a substantial body of research has identified natural compounds as promising starting points for new substances, particularly as protein kinase inhibitors. Among these natural compounds, examples like Emodin, Quercetin, and Curcumin have demonstrated utility in inhibiting JNK3 activity [55]. These compounds not only showcase the potential of natural resources in targeting critical pathways associated with diseases but also underscore the importance of further studies to unravel the full therapeutic potential of these compounds. As researchers continue to delve into the rich repository of natural compounds, new avenues for drug development and potential JNK3 inhibitors may emerge, offering innovative and safer solutions for various human diseases [56].

## Limitations

Despite the continuous discovery and synthesis of novel JNK3 inhibitors, the development of targeted and potent compounds remains a formidable challenge. This challenge stems from the unclear pathogenesis of many neurodegenerative and neurological diseases. Moreover, numerous JNK3 inhibitors, such as the nonspecific and low-toxicity JNK pan-inhibitor AS6001245, lack the required specificity for JNK3 inhibition, diminishing their therapeutic value and raising concerns about potential off-target effects on other JNK isoforms in peripheral tissues [57]. Some JNK3 inhibitors that necessitate oral administration exhibit inadequate bioavailability within the lesion, potentially requiring direct intra-lesion injections and escalating doses for therapeutic efficacy [58]. This not only escalates the treatment’s cost and complexity but also heightens the associated risks and patient discomfort [59]. Additionally, certain compounds that inhibit the phosphorylation of all JNK3 substrates, despite their neuroprotective potential, may inadvertently hasten the development of other diseases. For instance, mice treated with SP600125 during transient focal ischemia faced an increased risk of vascular infarction [60]. Notably, as of the present time, no JNK3 inhibitors have undergone validation in clinical trials for the treatment of human neurological disorders. Consequently, conducting clinical trials with JNK3 inhibitors remains a formidable task for scientists.

The impact of reversible and irreversible JNK3 inhibitors on other peripheral tissues is a crucial consideration. Understanding their potential effects beyond the targeted site is essential for assessing the overall safety profile. While specific details may vary among compounds, preclinical studies often explore potential off-target effects. Reversible inhibitors, competing with ATP for JNK3’s catalytic site, and irreversible inhibitors forming covalent bonds may exhibit different selectivity and interactions with related kinases. The risk of unintended interactions in peripheral tissues necessitates careful evaluation of the inhibitors’ specificity for JNK3 [61]. For example, the reversible inhibitor SP600125, a pan-JNK inhibitor, has shown potent inhibition of JNK1, JNK2, and JNK3, raising concerns about potential effects on other kinases in peripheral tissues [62]. Similarly, irreversible inhibitors like JNK-IN-8 and FMU200 require scrutiny for selectivity and potential cross-reactivity with kinases in non-target tissues [63]. Detailed pharmacological studies, including selectivity assays and in vivo investigations, are crucial for determining the impact of reversible and irreversible JNK3 inhibitors on peripheral tissues, ensuring their safety in broader physiological contexts.

## Conclusions and future directions

JNK3 is a heterodimer of JNK that is primarily expressed in the central nervous system. It is activated by a multilevel protein kinase phosphorylation cascade and plays a role in physiological processes such as the development of the brain, the formation of synapses, and the formation of memories. Since intracellular levels of JNK3 are associated with cognitive performance and serve as a biomarker of synaptic dysfunction, dysregulation of JNK3 activity is associated with several neurodegenerative diseases, such as stroke, Alzheimer’s disease, Parkinson’s disease, and epilepsy. As a result, JNK3 has emerged as a promising new therapeutic target for neurodegenerative conditions. This also means that JNK3 inhibitors have the potential to be developed as therapeutic tools for neurodegenerative diseases. Although, in recent years, many small molecule inhibitors of JNK3 have shown some neuroprotective effects in in vitro and in vivo experiments, they have not been able to be successfully applied in clinical treatment examples. Therefore, developing, and synthesizing JNK3 inhibitors with high selectivity, high inhibitory activity, high affinity, and high stability should be an essential path for future research. Natural resources have been used as a source of medical substances for thousands of years, and an astounding number of modern medications have been isolated from them, particularly plants. Many drugs are based on their use in a natural context, where they have a long and proven track record of treating a variety of illnesses, including cancer, stroke, and neurodegenerative disease [64]. Therefore, natural resources represent valuable lead compound libraries. With the development of computer technology, developing a paradigm incorporating structure-based drug design (SBDD) and Artificial Intelligence (AI) to generate lead compound/s from a large nature compound library through computational modeling before proceeding to animal studies may be a potential approach for screening JNK3 inhibitors. Additionally, developing dual-target or multi-target inhibitors that can simultaneously target other therapeutic targets is also a very promising direction for future research.

## Author contribution

Writing – Original Draft, Y.W., Z.G., and S.E.; Writing – Review & Editing, Y.W., Z.G., Y.Z., S.E., Z.C.X., and D.K. Writing – Revision, Y.W., and D.K.

## Data Availability

This does not apply to my study.

## Abbreviations

CNS: Central Nervous System
JNK: c-Jun N-terminal Kinase
MAPK: Mitogen-activated Protein Kinase
PI3K/AKT: Phosphatidylinositol 3-kinase and Akt protein kinase B
ERK: Extracellular signal-regulated protein kinase
TNF-α: Tumour necrosis factor
IL-6: Interleukin 6
ROS: Reactive oxygen species
OGD: Oxygen-glucose deprivation
Nrf2: Nuclear factor-erythroid factor 2-related factor 2
AP: Amyloid Plaques
NFT: Neurofibrillary Tangles
AD: Alzheimer’s Disease
ASK1: Apoptotic Signal-Regulated Kinase 1
PD: Parkinson’s disease
